# MTX-211 Inhibits GSH Synthesis through Keap1/NRF2/GCLM Axis and Exerts Antitumor Effects in Bladder Cancer

**DOI:** 10.3390/ijms24087608

**Published:** 2023-04-20

**Authors:** Bing Hu, Ru Chen, Ming Jiang, Situ Xiong, An Xie, Xiaoqiang Liu, Bin Fu

**Affiliations:** 1 Department of Urology, The First Affiliated Hospital of Nanchang University, 17 Yongwaizheng Street, Nanchang 330006, China; 2 Jiangxi Institute of Urology, Nanchang 430032, China

**Keywords:** MTX-211, GSH metabolism, proliferation, Keap1/NRF2/GCLM, bladder cancer

## Abstract

Globally, bladder cancer (BLCA) is still the leading cause of death in patients with tumors. The function and underlying mechanism of MTX-211, an EFGR and PI3K kinase inhibitor, have not been elucidated. This study examined the function of MTX-211 in BLCA cells using in vitro and in vivo assays. RNA sequencing, quantitative real-time polymerase chain reaction, Western blotting, co-immunoprecipitation, and immunofluorescence were performed to elucidate the underlying mechanism. Our observations revealed that MTX-211 has a time- and concentration-dependent inhibitory effect on bladder cancer cell proliferation. Flow cytometry analysis showed that cell apoptosis and G0/G1 cell cycle arrest were significantly induced by MTX-211. MTX-211 inhibited intracellular glutathione (GSH) metabolism, leading to a decrease in GSH levels and an increase in reactive oxygen species. GSH supplementation partly reversed the inhibitory effects of MTX-211. Further experiments verified that MTX-211 promoted NFR2 protein ubiquitinated degradation via facilitating the binding of Keap1 and NRF2, subsequently resulting in the downregulated expression of GCLM, which plays a vital role in GSH synthesis. This study provided evidence that MTX-211 effectively inhibited BLCA cell proliferation via depleting GSH levels through Keap1/NRF2/GCLM signaling pathway. Thus, MTX-211 could be a promising therapeutic agent for cancer.

## 1. Introduction

In the United States, the number of new bladder cancer (BLCA) cases and BLCA-related deaths in 2023 are projected to be 82,290 and 16,710, respectively. BLCA remains one of the top 10 malignancies that result in death among male patients [1,2]. The therapeutic strategies for BLCA are primarily surgical intervention, chemotherapy, radiotherapy, and immunotherapy. Treatments for BLCA are decided based on the pathological stage of the tumor [3,4]. Advancements in the field of BLCA therapy have not significantly contributed to enhancing the prognosis of patients with BLCA [5]. Thus, there is an urgent need to develop novel therapeutic targets or agents to improve patients’ clinical outcomes or augment the efficacy of current medications.

Cancer cells are usually subjected to elevated oxidative stress, derived from an overproduction of reactive oxygen species (ROS), as a result of genetic mutations, which can be induced by exposure to chemical agents [6], and abnormal growth patterns. The maintenance of the antioxidant glutathione (GSH) is, therefore, crucial for the survival and proliferation of cancer cells [7]. The GSH levels in breast, ovarian, head and neck, and lung cancers are reported to be higher than those in normal tissues [8]. GSH metabolism is also involved in tumorigenesis, progression, and metastasis [9]. Metabolomic analysis revealed that the metabolic pathways of GSH, purine, and thiamine were significantly altered in BLCA, with the GSH metabolic pathway exhibiting the most significant alterations [10]. The synthesis of GSH is regulated by several factors, including Glutamate-Cysteine ligase (GCL), which is comprised of Glutamate-Cysteine Ligase catalytic subunit (GCLC) and Glutamate-Cysteine Ligase modulatory subunit (GCLM), the activity of GCL, and the availability of cysteine [11,12]. GCLM contributes to the enhanced synthesis of GSH, which facilitates the development of tumors. The application of inhibitors of GSH synthesis before the onset of tumors holds the potential for effectively suppressing tumorigenesis [13].

NRF2 (Nuclear Factor Erythroid 2-related Factor 2), a hallmark of cancers, is a transcription factor involved in regulating the expression of genes involved in the cellular antioxidant response. Additionally, NRF2 is reported to be involved in the progression and dissemination of cancer, as well as conferring resistance to both chemotherapy and radiotherapy in the last decades [14,15,16,17,18]. Metabolic remodeling, including NRF2-mediated GSH synthesis, is a prevalent feature of tumors [14]. The biosynthesis of GSH can utilize glutamate as a substrate, under the regulation of NRF2 target genes, such as GCLC, GCLM, and Glutathione Synthase (GS). Previous functional enrichment analysis showed that GSH metabolism and the NRF2 pathway are correlated with the development of BLCA [19,20]. GCLM, a regulator of ferroptosis, is highly expressed in BLCA and promotes BLCA cell proliferation and migration. Additionally, GCLM upregulation is associated with unfavorable prognosis [21]. Therefore, modulating the metabolism of GSH via the NRF2/GCLM signaling pathway can be an effective therapeutic strategy for cancer or overcoming chemotherapy resistance.

MTX-211 (also called methanesulfonamide, N-[2-chloro-5-[4-[(3-chloro-4-fluorophenyl)amino]-6-quinazolinyl]-3-pyridinyl]) is a first-in-class dual inhibitor of epidermal growth factor receptor (EGFR) and phosphoinositide-3 kinase (PI3K) with a molecular weight of 478.33 and a chemical formula of C_20_H_14_Cl_2_FN_5_O_2_S. Maust reported that MTX-211 exhibited robust in vivo growth-inhibitory efficacy against BRAF-mutant and KRAS-mutant colorectal cancer models. Additionally, the survival rate of patients treated with the combination of MTX-211 and the MEK inhibitor trametinib was >400% when compared with that of patients treated with MTX-211 or trametinib alone [22]. Frankowski also demonstrated that the combination of MTX-211 and trametinib showed synergistic therapeutic effects on pancreatic cancer models. Compared to either agent alone, the combination therapy increased cell apoptosis and significantly impaired tumor growth in KRAS and p53 mutant (KPC) mice [23]. Taken together, MTX-211 could be a promising antitumor agent. However, its role in BLCA remains unknown. Thus, MTX-211 is a potential antitumor agent. In the present study, the efficacy of MTX-211 was examined in bladder cancer cells. We aimed to evaluate the impact of MTX-211 on various cellular processes, including bladder cancer cell proliferation, cell cycle regulation, and apoptosis. Furthermore, we sought to investigate the effect of MTX-211 on the Keap1/NRF2/GCLM signaling pathway and GSH metabolism.

## 2. Results

### 2.1. MTX-211 Inhibits Bladder Cancer Cell Proliferation in a Time- and Concentration-Dependent Manner

**Figure 1:**
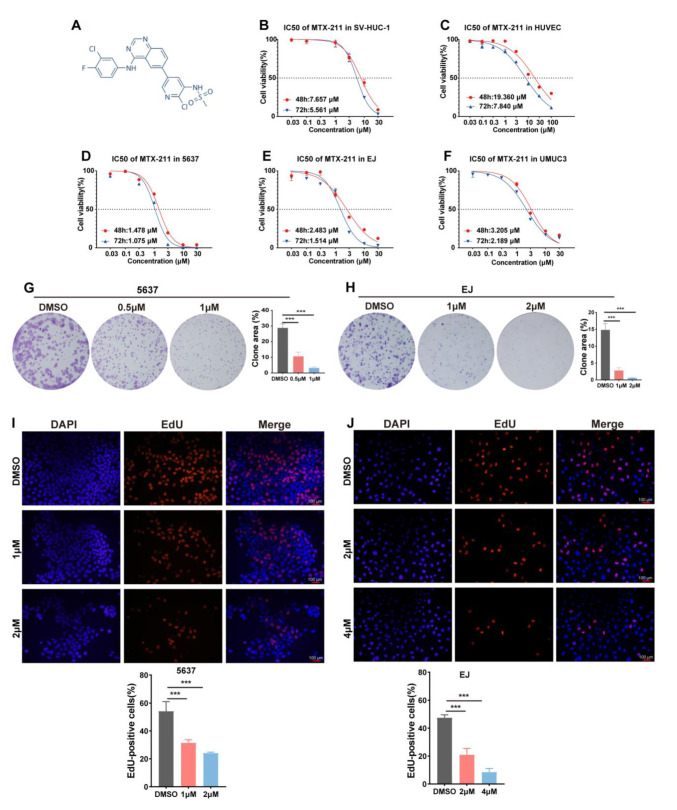
MTX-211 inhibits bladder cancer cell proliferation. (**A**) The structure of MTX-211. (**B**–**F**) The effects of treatment with different concentrations of MTX-211 for 48 and 72 h on the viability of bladder cancer cells. (**G**,**H**) The impact of MTX-211 on the colony formation ability of bladder cancer cells. (**I**,**J**) The effect of MTX-211 treatment for 48 h on cell proliferation was examined using the 5-ethynyl-2′-deoxyuridine (EdU) assay. *** *p* < 0.001. Scale bar: 100 μm.

The cytotoxicity effects of MTX-211 on BLCA cells were evaluated. The BLCA cells (5637, EJ, and UMUC3) and normal epithelial cells (SV-HUC-1 and HUVEC) were exposed to various concentrations (0, 0.03, 0.1, 0.3, 1, 3, 10, 30, and 100 μM) for different times (48 and 72 h). We applied the Cell Counting Kit-8 (CCK8) assay to examine the cell viability. As shown in Figure 1B–F, the cancer cell viability was significantly decreased in a time- and concentration-dependent manner. Meanwhile, the sensitivity of normal epithelial cells was less than that of the cancer cell lines. Next, the effect of MTX-211 on cell proliferation was examined using the colony formation and 5-ethynyl-2′-deoxyuridine (EdU) assays. Figure 1F,G show that MTX-211 dose-dependently inhibited the colony formation ability of cancer cells. Additionally, the EdU assay indicates that the proportion of EdU-positive cells decreased with the increased concentration (Figure 1I,J). These findings indicate that MTX-211 exhibits growth-inhibitory effects against BLCA cells in vitro.

### 2.2. MTX-211 Induces Apoptosis and Cell Cycle Arrest at GO/G1 Phase in BLCA Cells

**Figure 2:**
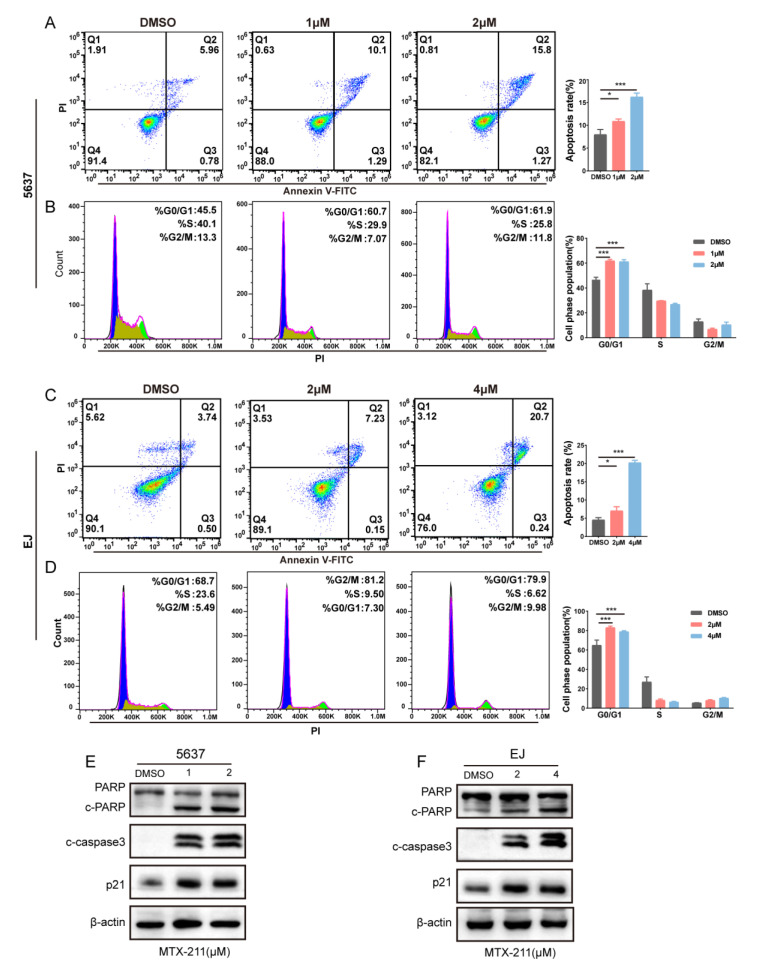
MTX-211 induces cell apoptosis and G0/G1 cell cycle arrest. (**A**–**D**) The effect of MTX-211 treatment for 48 h on the number of apoptotic cells and cells at different cell cycle phases in 5637 and EJ cells was analyzed using flow cytometry. (**E**,**F**) The protein expression of PARP, cleaved-caspase3, and p21 was detected using Western blotting. * *p* < 0.05, *** *p* < 0.001.

In order to elucidate the underlying mechanism of MTX-211 on cell death, we used flow cytometry analysis and Annexin V-FITC and PI double staining to detect the cell apoptosis level. After cells were treated with different concentrations for 48 h, the late apoptosis cell proportion was significantly increased in a dosage-dependent manner in both 5637 and EJ cells (Figure 2A,C). The expression levels of apoptosis-related proteins were examined using Western blotting. As illustrated in Figure 2E,F, the cleaved-PARP and cleaved-caspase 3 expression levels were upregulated in BLCA cells. Cell cycle dysfunction is one of the most important characteristics of tumorigenesis. Next, this study examined if MTX-211 exerted growth-inhibitory effects in BLCA cells by disrupting the cell cycle. PI staining revealed that MTX-211 treatment led to a G0/G1 phase cell ratio increase, while the proportion of S phase cells was considerably decreased in 5637 and EJ cancer cells (Figure 2B,D). Western blotting analysis showed that cell cycle inhibitor protein p21 levels were significantly elevated (Figure 2E,F). These data indicate that MTX-211 exerted growth-inhibitory effects in 5637 and EJ cells by inducing cell apoptosis and arresting the cell cycle at the G0/G1 phase.

### 2.3. Glutathione Metabolism Plays an Important Role in the Antitumor Effect of MTX-211

**Figure 3:**
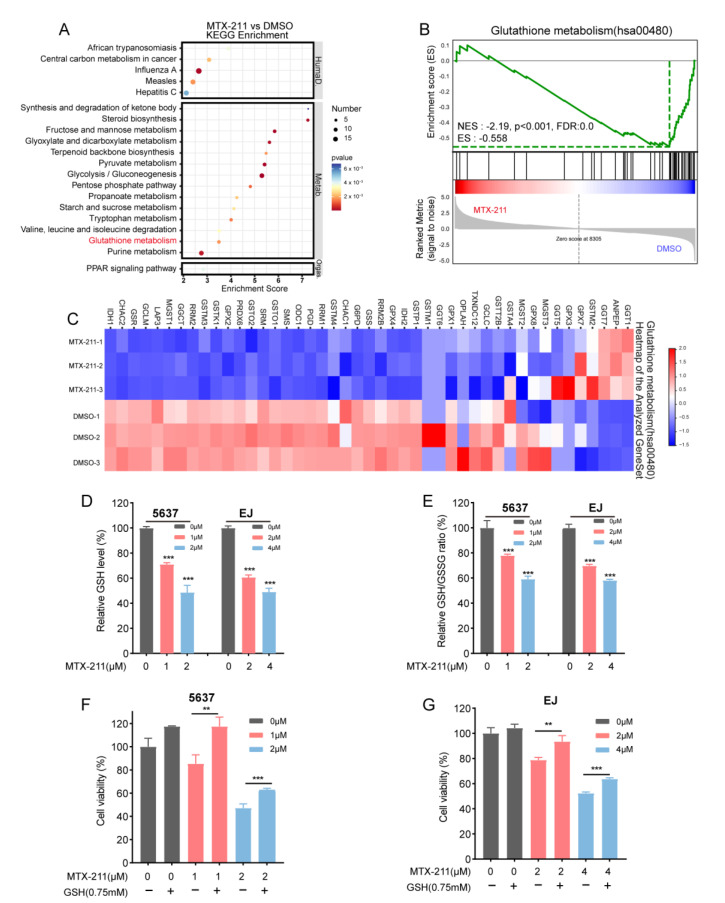
MTX-211 exerts antitumor effects by modulating glutathione (GSH) metabolism. (**A**,**B**) Kyoto Encyclopedia of Genes and Genomes (KEGG) pathway enrichment and Gene Set Enrichment Analysis (GSEA) between MTX-211- and DMSO-treated 5637 cells. (**C**) The heatmap of GSH metabolism gene set in MTX-211- and DMSO-treated groups. (**D**,**E**) The effect of treatment with different concentrations of MTX-211 for 48 h on the relative level of GSH and the relative ratio of GSH/GSH disulfide (GSSG) in 5637 and EJ cells. (**F**,**G**) The supplementation of GSH partially blocked the inhibitory effect of MTX-211 in 5637 and EJ cells. ** *p* < 0.01, *** *p* < 0.001.

Next, the antitumor mechanisms of MTX-211 were evaluated using RNA sequencing. The Kyoto Encyclopedia of Genes and Genomes (KEGG) pathway enrichment results revealed that MTX-211 treatment impaired GSH metabolism, which was also enriched in Gene Set Enrichment Analysis (GSEA) in 5637 cells (Figure 3A,B). The changes in the levels of GSH-metabolism-related genes after MTX-211 treatment were represented using a heatmap (Figure 3C). Then, the effects of MTX-211 on the intracellular GSH and GSH disulfide (GSSG) levels were examined in both cell lines. As shown in Figure 3D,E, MTX-211 treatment significantly decreased the GSH level and the ratio of GSH/GSSG in 5637 and EJ cells. Additionally, MTX-211 upregulated the ROS level in 5637 and EJ cells (Supplementary Materials Figure S1). Furthermore, GSH supplementation partially reversed the inhibitory effect of MTX-211 on cell proliferation. These findings indicate that MTX-211 exerted growth-inhibitory effects against BLCA cells partly through the reduction in intracellular GSH levels.

### 2.4. MTX-211 Promotes NRF2 Protein Ubiquitination and Degradation

**Figure 4:**
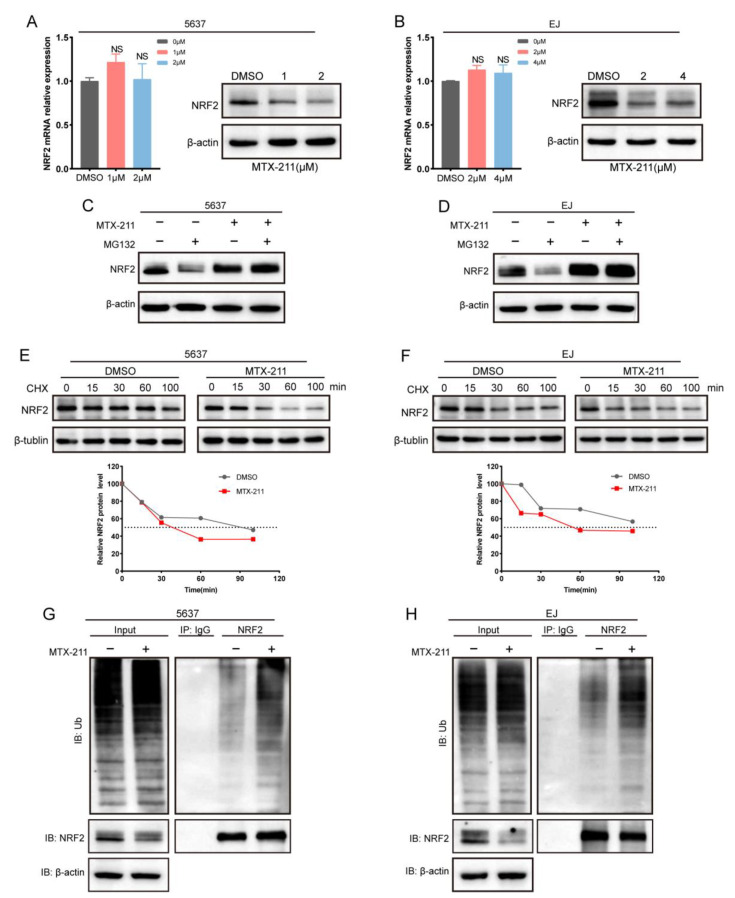
MTX-211 promotes NRF2 protein ubiquitinated degradation. (**A**,**B**) The effect of treatment with different concentrations of MTX-211 for 48 h on the mRNA and protein expression levels of NRF2 in 5637 and EJ cells. (**C**,**D**) Western blotting analysis of NRF2 in 5637 and EJ cells treated with DMSO or MTX-211 for 48 h, followed by treatment with or without MG132 (10 μM) for 4 h. (**E**,**F**) Following 48 h treatment with MTX-211 or DMSO, 5637 and EJ were harvested at the indicated time after treatment with cycloheximide (CHX: 20 μg/mL). Immunoblot of NRF2 was quantified. (**G**,**H**) The ubiquitination level of NRF2 in 5637 and EJ cells treated with MTX-211 for 48 h. IP: immunoprecipitation, NS: non-significance.

NRF2 is a critical intracellular transcription factor involved in anti-oxidative stress. The downstream target genes of NRF2, such as GCLM and GCLC, are involved in various biological processes, such as GSH synthesis. RNA sequencing analysis revealed that MTX-211 did not reduce NRF2 mRNA expression levels. Consistently, quantitative real-time polymerase chain reaction (qRT-PCR) analysis suggested that MTX-211 did not significantly affect the NRF2 mRNA expression in 5637 and EJ cells. However, MTX-211 significantly downregulated the NRF2 protein expression (Figure 4A,B). The major NRF2 post-translational modification of NRF2 is ubiquitination, which promotes proteasomal degradation. As shown in Figure 4C,D, treatment with MG132, a proteasome inhibitor, mitigated the MTX-211-mediated downregulation of NRF2 protein levels. In addition, MTX-211 accelerated NRF2 protein degradation in 5637 and EJ cells upon co-treatment with cycloheximide (CHX) (Figure 4E,F). The ubiquitination analysis revealed that MTX-211 treatment increased the ubiquitination levels of NRF2 (Figure 4G,H).

### 2.5. MTX-211 Decreased GSH Level via Keap1/NRF2/GCLM Signaling Pathway

**Figure 5:**
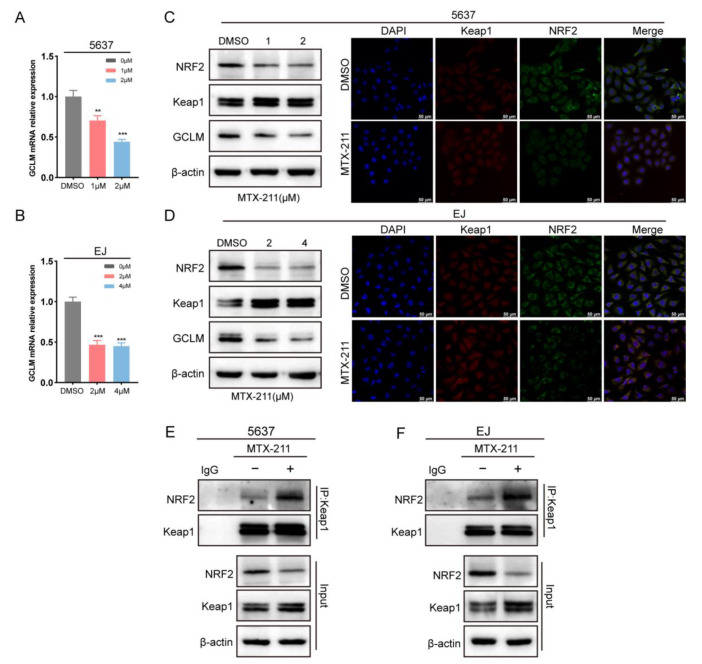
MTX-211 inhibits the Keap1/NRF2/GCLM signaling pathway. (**A**,**B**) The mRNA expression level of GCLM. (**C**,**D**) The expression levels of Keap1, NRF2, and GCLM were detected using Western blot and immunofluorescence analysis, which suggest Keap1 and NRF2 colocalized in 5637 and EJ cells. (**E**,**F**) Co-immunoprecipitation revealed that MTX-211 facilitated the binding of Keap1 to NRF2 proteins. ** *p* < 0.01, *** *p* < 0.001. Scale bar: 50 μm.

Keap1 serves as an E3 ubiquitin ligase and facilitates the degradation of the NRF2 protein. As shown in Figure 5C,D, MTX-211 upregulated the expression of Keap1 and downregulated the expression of NRF2. Immunofluorescence analysis revealed that Keap1 colocalized with NRF2 in both 5637 and EJ cells (Figure 5C,D), which was consistent with previous research findings. Additionally, the results of co-immunoprecipitation (COIP) indicate that MTX-211 treatment facilitated the binding of Keap1 to NRF2, leading to the ubiquitinated degradation of the NRF2 protein (Figure 5E,F). GCLM, a target gene of NRF2, encodes a component of the GCL enzyme that regulates the synthesis of GSH. The results from qRT-PCR (Figure 5A,B) and Western blotting (Figure 5C,D) analysis show that MTX-211 significantly decreased the GCLM expression levels in both 5637 and EJ cells. This suggests that MTX-211 can inhibit GSH synthesis by upregulating Keap1 expression and facilitating its binding to NRF2, leading to a reduction in NRF2 protein expression and, subsequently, decreased GCLM expression at both the transcriptional and translational levels.

### 2.6. MTX-211 Suppresses the Growth of BLCA Xenograft In Vivo

**Figure 6:**
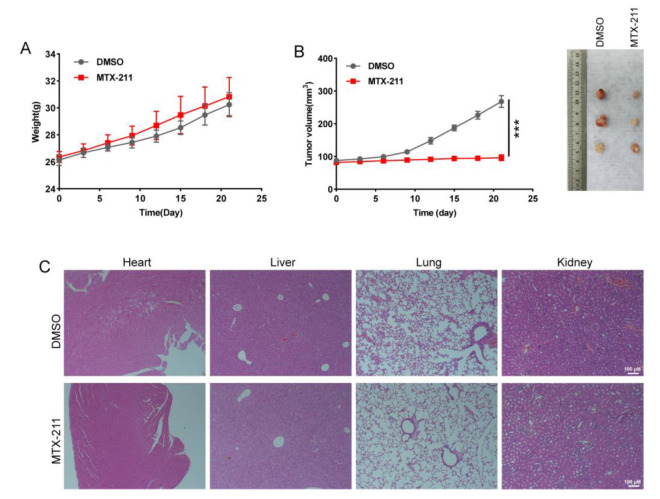
MTX-211 treatment inhibits the growth of the xenograft tumor. (**A**) The body weight of nude mice in DMSO- and MTX-211-treated groups. (**B**) The tumors were harvested and imaged at day 21 post-MTX-211 treatment. (**C**) Hematoxylin–eosin staining revealed that MTX-211 did not exert toxic effects on the organs of mice. *** *p* < 0.001. Scale bar: 100 μm.

To investigate the in vivo therapeutic effect of MTX-211, nude mice with xenograft tumors were randomly divided into DMSO- and MTX-211 (10 mg/kg bodyweight)-treated groups. The data suggest that MTX-211 treatment markedly decreased the growth of xenografts (Figure 6B). However, the body weight of nude mice was not statistically different between the two groups (Figure 6A). According to Hematoxylin–eosin staining analysis of the major organs revealed that MTX-211 did not induce evident tissue damage when compared with the DMSO group (Figure 6C).

## 3. Discussion

Previous studies have demonstrated the pivotal role of NRF2 and its regulatory effects on GSH metabolism in tumor development and therapeutic resistance [9,14]. Thus, the identification of known or novel drugs targeting NRF2 and GSH metabolism holds significant promise in improving patient prognosis and augmenting the efficacy of existing therapeutic interventions. The effect of MTX-211, a dual inhibitor of EGFR and PI3K kinase, on BLCA has not yet been previously reported. This study demonstrates that MTX-211 exerts a potent inhibitory effect on GSH synthesis via the Keap1/NRF2/GCLM signaling pathway in BLCA cells and results in suppressing proliferation, upregulating apoptosis, and inducing cell cycle arrest at the G0/G1 phase. Additionally, the results of in vivo experiments demonstrate that MTX-211 effectively suppressed the growth of xenograft tumors. Thus, this study offers a promising therapeutic strategy for the treatment of BLCA.

The Ba/sq (basal/squamous) subtype, which accounts for about 35% of all cases of muscle-invasive BLCA, is characterized by EGFR overexpression [24]. A representative of this subtype is the 5637 cell line [25]. Rose et al., demonstrated a significant upregulation of EGFR at both the mRNA and protein levels in Ba/sq BLCA [26]. Rebouissou et al., reported abnormal activation of the EGFR pathway in the Ba/sq subtype resulting from autocrine signaling and upregulation of EGFR. Additionally, the authors further observed that EGFR inhibitors exerted potent therapeutic effects in a basal BLCA mouse model [25]. Furthermore, EGFR can activate the PI3K/AKT signaling pathway by extracellular signals. However, the dysregulation of the PI3K/AKT signaling pathway is reported to be a key factor in the tumorigenesis of BLCA, with approximately 40% of patients with BLCA exhibiting disrupted PI3K/AKT signaling [27]. Chen et al., demonstrated the effectiveness of Voacamine in suppressing tumor cell proliferation by inhibiting EGFR and its downstream PI3K/AKT signaling pathway, inducing cell cycle arrest, promoting apoptosis, and increasing cleaved-caspase3 expression [28]. Our study shows that MTX-211 exerted a potent anti-proliferation effect against 5637 and EJ cells by inducing cell cycle arrest and apoptosis.

Although MTX-211 functions as an inhibitor of EGFR and PI3K kinases, the therapeutic agent’s mechanism frequently encompasses modifications of multiple signaling pathways. In 2011, Hanahan et al., introduced the reprogramming of cellular metabolism as a novel hallmark of cancer, and GSH metabolism is implicated in the biological processes of tumorigenesis, progression, metastasis, and drug resistance [9,29]. To explore its other underlying mechanism, through RNA sequencing analysis, we discovered that MTX-211 modulates intracellular GSH metabolism, and subsequent validation experiments confirmed a reduction in cellular GSH levels concomitant with an upregulation in ROS levels. On the one hand, GSH is widely distributed within cellular structures, and its elevated concentrations are essential for cells to advance from the G1 phase to the S phase. Conversely, an increase in GSSG leads to prolonged arrest in the G1 phase, resulting in cell death [30,31]. GSH protects the integrity of DNA by providing the appropriate redox environment for replication. Additionally, GSH is a key factor in epigenetic events that are essential for the regulation of cell proliferation and cellular resistance to apoptosis [31,32]. Therefore, disruptions in GSH metabolic processes have been shown to induce cell apoptosis. This indicates that GSH metabolism is a potential mechanism for restricting tumor growth, which can be potentiated with the administration of the GSH booster baicalein [33,34]. On the other hand, excessive ROS, the by-product of oxygen consumption and cellular metabolism, could promote oxidative-stress-induced cancer cell death [35,36]. The activation of caspases is considered to be one of the most extensively researched hallmarks of apoptosis, which ultimately leads to the cleavage of PARP, and cell death [37]. Elevated ROS can directly activate caspase function and contribute to the induction of late-stage apoptosis [35,36]. Late-stage apoptosis often leads to cellular damage that cannot be repaired and results in cell death. Hence, downregulated GSH and upregulated ROS can trigger cell death. This study reveals that MTX-211 could potently inhibit cell proliferation and induce late-stage apoptosis and cell cycle arrest at the G0/G1 stage via depleting cellular GSH and elevating ROS levels.

To elucidate the underlying mechanism of MTX-211-induced GSH depletion, we further verified that MTX-211 inhibited the Keap1/NRF2/GCLM signaling pathway. Previous studies have suggested that NRF2 and its target genes are the key regulators in cellular antioxidant stress and energy metabolism processes, including the Warburg effect and GSH synthesis [14,38]. NRF2 protein expression is regulated by an adapter protein, identified as Keap1, BTRC, and SYVN1, which serves as a bridge to the E3 ubiquitin ligase complex, resulting in degradation by the proteasome [39,40,41]. Under physiological conditions, elevated ROS levels impair the function of Keap1, promoting NRF2 nuclear translocation and the activation of its target gene transcription [38]. As the Keap1/NRF2 axis is disrupted in various cancers, it is a potential therapeutic target for cancer and chemoresistance [42,43]. Previous research has reported that the activation of the NRF2 signaling pathway by p62 occurs through the inhibition of Keap1, leading to increased expression of GCLC. This, in turn, results in an elevation of GSH levels and decreased ROS, promoting the proliferation of BLCA cells [44]. GCLM is also considered an oncogene in BLCA, which correlates with unfavorable prognosis and accelerated cell proliferation [21,45]. Our findings indicate that MTX-211 reduces the expression of NRF2 through destabilization rather than affecting its translation. COIP analysis suggests that the binding of Keap1 and NRF2 was increased after MTX-211 treatment. Consequently, GCLM expression was decreased in both transcription and translation levels. Taken together, MTX-211 promoted Keap1-mediated NRF2 ubiquitinated degradation, resulting in the downregulation of GCLM.

Cisplatin (CDDP), which is a first-line used chemotherapeutic agent for BLCA, exerts antitumor effects by increasing intracellular ROS levels and inducing oxidative stress [46]. Resistance to CDDP treatment is closely associated with increased intracellular GSH levels [47]. The GSH metabolic pathway is highly activated in a model of lung cancer brain metastasis when compared with that in primary tumors, leading to platinum-based chemotherapy resistance in metastases [48]. Increased GSH levels also contribute to CDDP resistance in head and neck tumors [49]. Sang et al., demonstrated that Jolkinolide B inhibited GSH synthesis, increased intracellular ROS levels, and activated endoplasmic reticulum stress-induced apoptosis in BLCA, which significantly enhanced the sensitivity of CDDP-resistant T24 cell lines to CDDP [50]. Consequently, MTX-211 holds great potential as a therapeutic agent to enhance the effectiveness of CDDP or reverse the resistance to CDDP, making it a promising avenue for future research. This research has several limitations that warrant further investigation. First of all, while elevated levels of GSH have been reported in various tumors, the concentration of GSH in BLCA relative to adjacent tissue remains unclear. Furthermore, considering the multiple pathways in NRF2 ubiquitinated degradation, whether MTX-211 destabilization of the NRF2 protein depends on Keap1 needs to be further clarified. If MTX-211 promotes NRF2 degradation through Keap1, it may hinder the therapeutic application in Keap1-mutated cancers, such as non-small cell lung cancer.

## 4. Materials and Methods

### 4.1. Reagents and Antibodies

MTX-211 (S6541) was purchased from Selleck. L-Glutathione reduced (GSH) (HY-D0187) was obtained from MedChem Express (Princeton, NJ, USA). The antibodies used in the following experiments were anti-PARP (9532S, 1:1000), anti-caspase3 (9662S, 1:1000), anti-p21 (2947S, 1:1000), and anti-ubiquitin (3936S, 1:1000) obtained from Cell Signaling Technology; anti-keap1 (sc-365626, 1:1000), anti-GCLM (sc-55586, 1:1000) were from Santa Cruz; and anti-NRF2 (T55136, Abmart, 1:1000), anti-keap1 (10503-2-AP, Proteintech, 1:2000), and anti-p62 (ab109012, Abcam, 1:10,000). Anti-rabbit IgG (DyLight 488, FD0136) and anti-mouse IgG (DyLight 649, FD0144) were from Hangzhou Fude biological technology (Fdbio science, Hangzhou, China).

### 4.2. Cell Culture

BLCA cell lines (5637, EJ, UMUC3), HUVEC, and SV-HUC-1 were bought from the cell bank of Type Culture Collection of Chinese Academy of Science (Shanghai, China). HUVEC, (5637 and EJ), UMUC3, and SV-HUC-1 cells were cultured in DMEM, RPIM-1640, MEM, and F12K containing 10% FBS and 1% penicillin/streptomycin at 37 °C and 5% CO_2_. Mycoplasma contamination testing results showed negative for the used cell lines.

### 4.3. Cytotoxic Activity Assays

To explore MTX-211′s cytotoxic effect on human normal epithelial cells (HUVEC and SV-HUC-1) and bladder cancer cell lines (5637, EJ, and UMUC3), 5000 cells were seeded into 96-well plates and treated with various concentrations (0, 0.03, 0.1, 0.3, 1, 3, 10, 30, 100 μM) of MTX-211 during the next day and incubated for 48 and 72 h. Then, cell viability was detected using a CCK8 Kit (Keygen, Nanjing, China). Briefly, cells were incubated with the 100 μL diluted CCK8 solution (10% in serum-free medium) for 2 h at 37 °C. The absorbance of the reaction mixture was measured at 450 nm. The half-maximal inhibitory concentration (IC50) of MTX-211 was calculated for each cell type based on cell viability using GraphPad Prism 7.0.

### 4.4. Cell Proliferation Assays

To examine the effect of MTX-211 on cell proliferation, 5637 or EJ cells (1000 cells/well) were seeded into 6-well plates and cultured overnight. Next, the cells were incubated with different concentrations of MTX-211 for 7–14 days, then the cells were fixed and stained by 4% paraformaldehyde (20 min) and 0.5% crystal violet (20 min) at room temperature, respectively. The colony area was quantified using ImageJ (v 1.52p). Additionally, we also evaluated its effects on cell proliferation using an EdU assay kit (C10310-1, Ribo Bio., Guangzhou, China) under the manufacturer’s instructions. Briefly, 5637 and EJ cells (5000 cells/well) were seeded in a 96-well plate and incubated overnight for attachment. After 48 h treatment of MTX-211, cells were incubated with 50 μM EdU for 2 h at 37 °C, 4% paraformaldehyde for 30 min, 2 mg/mL glycine for 5 min, 0.5% TritonX-100 for 10 min at room temperature, respectively. Subsequently, the cells were stained with 1× Apollo staining reaction for 30 min, washed with 0.5% TritonX-100 for 10 min, and, lastly, stained with DAPI for 30 min at room temperature. The images were acquired under the Cy3 and DAPI channels of a fluorescent inverted microscope (Olympus, Tokyo, Japan). The proportion of EdU-positive cells was quantified using ImageJ.

### 4.5. Cell Apoptosis and Cycle Assays

5637 and EJ cells were seeded into 6-well plates. After being treated with MTX-211 for 48 h, cells were enzymatically dissociated and washed three times with cold PBS. To evaluate apoptosis events, the cells were stained with a combination of 5 μL PI (20 μg/mL) and 5 μL Annexin V/FITC (50 μg/mL) (FA101-01, Transgen, Beijing, China) in PBS for 15 min at room temperature in the dark [51]. On the other hand, for the analysis of cell cycle, cells were fixed in 75% alcohol overnight at 4 °C. Then, after incubating with 20 μL RNase A for 30 min at 37 °C, cells were stained with 400 μL PI (100 μg/mL) (BB-4104, Bestbio, Shanghai, China) in PBS for 30 min in the dark at 37 °C the next day. Subsequently, both sets of stained cells were analyzed using flow cytometry, and the results were processed and analyzed using Flowjo software (v 10.4).

### 4.6. RNA Sequence Anaylsis

The 5637 cell line was treated with MTX-211 for 48 h and then harvested for RNA sequencing analysis. The sequencing was conducted by OE Biomed Tech Co., Ltd. (Shanghai, China), which supplied the gene expression profiles and relevant pathway analysis outcomes. The raw data NCBI accession number is SRR24100329-34. The details of the sequencing and analysis protocols are provided below.

Under the manufacturer’s instruction, total RNA was extracted using the TRIzol reagent (Invitrogen, Carlsbad, CA, USA). A NanoDrop 2000 spectrophotometer (Thermo Scientific, Waltham, MA, USA) was used to assess RNA purity and quantification, and an Agilent 2100 Bioanalyzer (Agilent Technologies, Santa Clara, CA, USA) was used for RNA integrity. Then, the libraries were constructed using VAHTS Universal V6 RNA-seq Library Prep Kit according to the manufacturer’s protocol. The libraries were sequenced on an Illumina Novaseq 6000 platform, and 150 bp paired-end reads were generated. The results showed that about 62–65 M raw reads for each sample were generated. Raw reads of fastq format were firstly processed using fastp [52]^,^ and the low-quality reads were removed to obtain the clean reads. Then, about 46–50 M clean reads for each sample were retained for subsequent analyses. The clean reads were mapped to Homo sapiens using HISAT2 [53]. FPKM [54] of each gene was calculated, and the read counts of each gene were obtained by HTSeq-count [55].

The DESeq2 was used to perform differential expression analysis [56]. Q value <0.05 and fold change >2 or fold change <0.5 was set as the threshold for significant differential expression genes (DEGs). Hierarchical cluster analysis of DEGs, the screening of significantly enriched terms in KEGG [57] pathway analysis of DEGs, and the drawing of the bubble diagram of the significant enrichment term were performed using R (v 3.2.0). GSEA was performed using GSEA software (v 4.1.0) [58]. The analysis used a predefined gene set, and the genes were ranked according to the degree of differential expression in the two types of samples. Then, it was tested whether the predefined gene set was enriched at the top or bottom of the ranking list.

### 4.7. qRT-PCR

Following 48 h of MTX-211 treatment, cellular RNA of 5637 and EJ cells was extracted using Trizol reagent, and then, we synthesized cDNA using a reverse transcription kit (KR116, TIANGEN BIOTECH, Beijing, China). The mRNA expression was quantitative in the PCR system (Applied Biosystems, Waltham, MA, USA) using ACTB as an internal reference, and the cycling conditions were as follows: denaturation at 95 °C for 1 min, amplification at 95 °C for 15 s and 60 °C for 30 s for 40 cycles. The primers used for amplification were as follows: ACTB: F: 5′-TCTCCCAAGTCCACACAGG-3′ and R: 5′-GGCACGAAGGCTCATCA-3′, NRF2: F: 5′-TTCTCCCAATTCAGCCAGCC-3′ and R: 5′-GGGAATGTCTGCGCCAAAAG-3′, GCLM: F: 5′-CTCCTGCTGTGTGATGCCA-3′ and R: 5′-CTCGTGCGCTTGAATGTCAG-3′.

### 4.8. ROS and GSH/GSSG Assays

EJ and 5637 cells were cultured in 6-well plates overnight. Then, using fresh medium containing the indicated concentration of MTX-211, we replaced the growth medium and cultured for 48 h. An ROS assay kit (S0033S, Beyotime, Shanghai, China) was used to detect the intracellular ROS levels, and the protocol was as follows: The treated cells were incubated with 1.5 mL DCFH-DA (5 μM) for 20 min in 37 °C. Then, the serum-free medium was used to wash the cells three times, and the images were acquired using a fluorescent inverted microscope (Olympus, Tokyo, Japan). The levels of GSH and GSSG (oxidized GSH) in 5637 and EJ cells were determined using a GSH and GSSG assay kit (A006-2-1, Nanjing Jiancheng Bioengineering Institute, China, and S0053, Beyotime, Shanghai, China), respectively, as per the manufacturer’s instruction.

### 4.9. Western Blotting

Following 48 h of exposure to MTX-211 in 6 cm plates, 5637 and EJ cells were harvested with 150 μL cell lysis buffer for Western and IP lysates (P0013, Beyotime, Shanghai, China) containing protease inhibitor on ice for 30 min and, subsequently, centrifuged at 12,000 rpm for 20 min at 4 °C. Protein was quantified using a BCA assay kit (PA115, TIANGEN BIOTECH, Beijing, China) and subjected to electrophoresis. The proteins were separated by SDS-PAGE and then transferred to PVDF membranes. The membranes were blocked with 5% skimmed milk at room temperature for 2 h and then incubated overnight at 4 °C with primary antibodies. Subsequently, the membranes were washed thrice with TBST and then incubated for 1 h at room temperature with HRP-conjugated secondary antibodies (1:4000). Finally, the membranes were washed five times with TBST, and the protein was detected using an ECL kit (NCM Biotech, Suzhou, China).

### 4.10. Co-Immunoprecipitation

The ubiquitination level of NRF2 protein and the binding of Keap1 and NRF2 were detected using COIP assay. To precipitate the target protein, 500 μL lysate of 5637 or EJ cells treated with MTX-211 for 48 h was incubated with 5 μL of primary antibody overnight at 4 °C. On the following day, the protein–antibody complex was incubated with 25 μL magnetic beads (HY-K0202, MedChem Express, Monmouth Junction, NJ, USA) with rotation at 4 °C for 3 h. Subsequently, the magnetic beads were separated from the supernatant using magnetic separation and washed 5 times with PBS. The immunoprecipitated proteins were then extracted by boiling the magnetic beads in SDS-PAGE loading buffer for 10 min and, subsequently, detected through Western blotting.

### 4.11. Immunofluorescence Analysis

The 5637 and EJ cells were cultured on 24-well plates with coverslips and exposed to MTX-211 for 48 h. Cells were fixed with 4% paraformaldehyde for 20 min, rinsed three times with PBST, permeabilizated with 0.5% Triton-X100 for 20 min, and blocked with 5% skim milk for 2 h at room temperature. Subsequently, the cells were incubated with 200 μL primary antibodies (anti-p62 (1:200), anti-Keap1 (1:200), and anti-NRF2 (1:200)) at 4 °C overnight. Next, the cells were washed thrice with PBST and then incubated with corresponding DyLight-conjugated secondary antibody (1:400) in the dark at room temperature for 2 h. After washing five times with PBST, the cells were stained using DAPI. The results were analyzed using confocal microscopy (Leica, Wetzlar, Germany).

### 4.12. Tumor Xenograft Models

Tumor xenograft models were established using 5-week-old male Balb/c nude mice. The right axillary region of nude mice was subcutaneously injected with 5637 cells (approximately 1 × 10^7^). When the xenografted tumor volume (1/2 × length × width^2^) reached approximately 100 mm^3^, the mice were randomly allocated into the MTX-211-treated and DMSO-treated groups. MTX-211 was administered at a dose of 10 mg/kg body weight once every two days. The tumor size was measured once every three days. At day 21 post-administration, the tumors were excised for further analysis. This animal study was approved by the Ethics Committee of the First Affiliated Hospital of Nanchang University (ethical number: CDYFY-IACUC-202208QR005).

### 4.13. Statistical Analysis

All statistical analyses were performed using GraphPad Prism 7.0 (GraphPad Software Inc., San Diego, CA, USA). The data were represented as mean ± standard deviation. Means between two groups were compared using Student’s *t*-test, whereas those between multiple groups were compared using one/two-way ANOVA. The significance level was set at *p* < 0.05.

## 5. Conclusions

In conclusion, MTX-211 exerts antitumor effects against BLCA cells by inhibiting proliferation and inducing cell death and cell cycle arrest. Mechanically, MTX-211 exerted antitumor effects by upregulating Keap1 expression, facilitating the binding of Keap1 to NRF2, thereby resulting in the ubiquitin-mediated degradation of NRF2. The decreased expression of NRF2 induced the downregulation of GCLM, which contributed to the inhibition of GSH synthesis.

Further studies are needed to fully elucidate the effects of MTX-211 and optimize its use in clinical applications. The findings of this study provide a promising new avenue for research in the field of cancer treatment and highlight the importance of targeting GSH in the development of novel therapeutic strategies.

## Data Availability

Datasets from this study can be obtained from the authors upon reasonable request.

## Supplementary Materials

The supporting information can be downloaded at: https://www.mdpi.com/article/10.3390/ijms24087608/s1.
