# Supplementary material for: MTX-211 Inhibits GSH Synthesis through Keap1/NRF2/GCLM Axis and Exerts Antitumor Effects in Bladder Cancer
Source: Int J Mol Sci. 2023 Apr 20;24(8):7608. doi: 10.3390/ijms24087608 (PMC10142351; doi:10.3390/ijms24087608)
Supplement: Supplementary file 1 [file ijms-24-07608-s001.zip › ijms-2249312-supplementary.pdf]

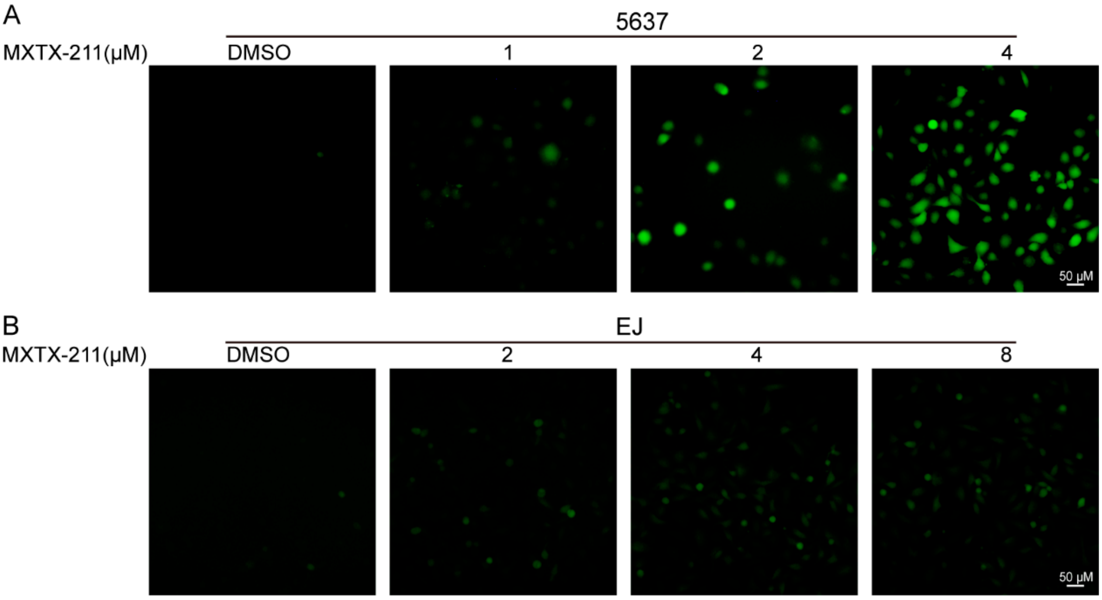

Figure S1. ROS level of in 5637 and EJ cells after treated with MTX-211 for 48 h. (A-B) ROS assay kit was used to detect ROS levels in 5637 and EJ cells following 48 h MTX-211 treatment. Scale bar: 50 μm.
